# Round about the Asylums—XI

**Published:** 1888-08-04

**Authors:** 


### Round About the Asylums.—XI.

By a Medical Superintendent.

### NEWCASTLE-UPON-TYNE ASYLUM.

Three hundred and nineteen patients were resident at the close of the year. There were 119 admissions—a high number for the size of the asylum—and the recoveries were 43·4 per cent.; 29 patients died, being at the rate of 9·5 per cent. on the average number resident; eight of the deaths were from general paralysis. The causes of insanity in those admitted do not differ much from those mentioned when speaking of other asylums. Hereditary predisposition comes first by a long way, and then drink. Dr. Wickham says: "If those who take an interest in social problems would apply themselves as energetically to bringing public opinion to bear on the prevention of unsuitable marriages as they do in other matters, there would be less of an outcry from the more moderate about interference with the liberty of the subject, and they would be working much nearer the fountain head than they generally are." The charge for Newcastle patients is 9*s.* 11*d.*, for out-county cases 14*s.*, and for private patients 16*s.* This is the twenty-third year of the existence of the asylum, and considerable additions have lately been built to the fabric.

### ROYAL ASYLUM, ABERDEEN.

This asylum contains 575 patients, and nearly 200 of these are private cases. The recoveries were 45 per cent. on the admissions, and the deaths 10·3 on the average number resident. Some of the private patients are in detached buildings, and the Commissioners say these "are treated with great kindness and in a very able manner." The main building is overcrowded, but the question of enlarging it is under consideration. Private patients pay from £28 up to £210 a-year, and the charge for Aberdeenshire paupers is £26. It has always seemed to us very strange that our English county asylums do not follow the example of these royal asylums, and provide separate accommodation for private patients. There cannot be a doubt that such adjuncts would be remunerative, and they would soon solve the private asylum question. Perhaps the permissive clauses of the Lunacy Bill may be acted on, but, inasmuch as most asylums do not keep pace with the demand for pauper accommodation, we fear these clauses will remain a dead letter. In spite of emergency clauses and royal asylums, Scotland seems as backward as England in obtaining early treatment. "It is a great disadvantage that mental disorders are not looked upon more in the same light as ordinary physical diseases, and that so much reluctance is felt in placing relations in an asylum, which ought to be regarded as an ordinary hospital. Many of the patients, and particularly private ones, have incurability stamped upon them on admission, and it is only as a last resource that parties will send their relatives for asylum treatment, which, in many cases, should have been done months previously."

### STAFFORD COUNTY ASYLUM AT LICHFIELD.

This asylum is 23 years old, and it had at the close of its first year of existence only 195 patients, whereas it now contains 554. During 1887, 117 were admitted, 119 were discharged, and 57 died. The recoveries were 38·46 on the admissions, and the deaths were 10·17 on the average number resident. We are pleased to find the following words in the medical superintendent's report, and we hope they will find an echo in the hearts of other superintendents, some of whom are too fond of advising that such cases as he refers to should not be sent to the asylum. "The admissions from workhouses of helpless chronic cases requiring much special care were not few. Complaint is frequently made that asylums should be hampered with these cases, but for my part I rejoice to think that the means placed at our disposal for nursing and caring for such poor and afflicted sufferers are happily and beneficially employed in lightening the burden of their troubles, and soothing their last days in a world that has not, as a rule, proved a very tender step-mother to many of the class from which these patients are usually derived." The weekly cost is 8*s.* 5¼*d.* There are only two private patients who pay 14*s.* a week each. A new recreation hall is being built, and there would seem to have been a great many minor improvements and alterations carried out during the year. The Commissioners say: "The wards and dormitories were in good order, the supply of books and papers in the wards sufficient, and the rooms looked bright and cheerful." So far as we have yet gone, Dr. Spencer is the only superintendent bold enough to quote poetry in his report.

### GLOUCESTER COUNTY ASYLUMS.

There are two asylums, both close to the City of Gloucester and about one mile apart from each other. They are under the same management. The number of patients at the close of the year was 933. The recoveries stand at 44 per cent. on the admissions, and the deaths at 9·97 on the average number resident. The death rate is much higher in the old asylum than in the new one; but it is stated that epileptics and paralytics are not sent to the latter, which partly at least accounts for the difference.

The average weekly cost per head is 8*s.* 6⅝*d.* There were three criminal patients, and seven private cases. The latter pay 14*s.* and 15*s.* a week. All sorts and conditions of men were represented in the admissions. We notice among them a scavenger and a bishop, a mother's help and a music-key filer, four governesses and six mill hands, an authoress and a costermonger. The commissioners say, "the patients' clothing is suitable for the season of the year. The dietary is satisfactory, and the general health seems to be good."

### LANCASTER COUNTY ASYLUM AT RAINHILL.

Like the Devizes Asylum, this is also in its thirty-seventh year, and its numbers have increased from 296 to 852. The recoveries were 22·63 on the admissions, and the deaths were 9·31 on the average number resident. The weekly rate is 9*s.* 3*d.* We extract the following from the medical superintendent's report: "Under the operation of recent legislation, persons having become insane whilst undergoing sentences of penal servitude are, at the end of their sentences, no longer technically 'criminal lunatics,' and are therefore transferred as ordinary pauper patients to county asylums; but they are a far more dangerous class than those to whom the term is now legally applicable; and if I might devise a name for them, I would call them 'lunatic criminals'—implying that they were 'criminals' first and 'lunatics' afterwards. It is by this class that murderous assaults in asylums are generally committed." This is perfectly true, and yet our county asylums have to be turned into prisons and our attendants into gaolers, because the Government will not build a suitable asylum for such cases. A large annexe for 1,000 patients was opened during the year, and we do not wonder that Dr. Rogers experienced difficulty in finding suitable attendants. We are pleased to find that he does not engage those who have served in other asylums, and if this rule be adhered to, there cannot be a doubt that the raw material will be forthcoming, provided sufficient wages be given. The Commissioners remark that the case books are very well and fully entered up. The committee acknowledge their indebtedness to Dr. Rogers for his able management and supervision of the whole establishment.

